# Severe Pulmonary Infection in a 20-Month-Old Female

**DOI:** 10.1155/2020/7301617

**Published:** 2020-02-12

**Authors:** Yasmeen Mann, Paul Zeller, Kristen Carrillo-Kappus, Melissa Victor, Mary Moore

**Affiliations:** ^1^Central Michigan University College of Medicine, Mount Pleasant, MI 48858, USA; ^2^Department of Family Medicine, Central Michigan University College of Medicine, Saginaw, MI 48602, USA; ^3^Department of Pediatrics, Central Michigan University Health, Saginaw, MI 48602, USA

## Abstract

Community-Acquired Pneumonia (CAP) is a common reason for hospitalization of a pediatric patient. We report a 20-month-old female admitted for suspected CAP. History included a week-long cough, fever, dyspnea, single occurrence of seizure-like activity, and a sick contact. Initial chest X-ray (CXR) showed left lower lobe pneumonia and parapneumonic effusion with a complex left pleural effusion. Ultrasound findings prompted the need for contrast-enhanced computed tomography (CT) of the chest. Contrast-enhanced CT of the chest confirmed a large pleural effusion with major atelectasis and mediastinal shift. The patient was treated with empiric antibiotics, video-assisted thoracoscopic surgical (VATS) decortication of empyema, and chest tube placement. Due to intraoperative complications, the VATS decortication was aborted and patient was transferred to the pediatric intensive care unit (PICU). A thoracentesis with culture failed to isolate a bacterial organism. Dexamethasone was started after repeat CXR showed persistent infiltrate. Subsequent contrast-enhanced CT of the chest showed a large collection of air and persistent consolidation. The patient received repeat VATS decortication and reinsertion of a chest tube. Repeat pleural fluid cultures failed to isolate a bacterial organism. Infectious disease (ID) consult recommended linezolid 140 mg Q8H for 4 weeks. Seven days after second VATS, a respiratory pathogen panel was positive for rhinovirus/enterovirus. With resolution of leukocytosis and clinical improvement, the patient was discharged with the chest tube in place and pediatric surgery outpatient follow-up. After three months, sequalae from both the infection and interventions presented .

## 1. Introduction

Community-Acquired Pneumonia (CAP) is one of the most common reasons for hospitalization in the pediatric population [1]. Occasionally, the pneumonia may be severe enough to be complicated by an empyema. Common causative organisms include *Streptococcus pneumoniae*, *Staphylococcus aureus*, and *Streptococcus pyogenes*. Viral causes, while not uncommon, tend to occur less frequently. In some cases, both a viral and bacterial source contribute to the development of CAP. In these instances, it is difficult to completely rule in or rule out every organism due to limitations in isolation of the causative organism and sensitivity and specificity of laboratory testing. In this report, we describe an interesting case of CAP complicated by empyema in an otherwise healthy pediatric patient and the hospital course that followed. While we were unable to confidently identify the causative microorganism, her case heighted our suspicion of viral causes of CAP, still emphasizing the importance of following culture results while treating complex cases of infection.

## 2. Case Presentation

A 20-month-old Caucasian female presented to the Emergency Department (ED) with complaint of a week-long cough, fever, difficulty breathing, and a single instance of apparent seizure-like activity that resolved. She was seen at an urgent care 7 days prior to ED presentation and given prescriptions for albuterol and oseltamivir for suspicion of Influenza A. The seizure-like activity occurred 5 days later, prompting a visit to her primary care provider (PCP). Caregivers refused Emergency Medical Services (EMS) transport at the time of seizure-like activity in lieu of scheduling a PCP visit. Her PCP visit occurred 2 days prior to ED presentation. Her PCP advised to discontinue albuterol.

The patient continued to have a nonproductive cough, fevers with a TMax of 105.0 Fahrenheit (40.5°Celsius) treated with Tylenol, 4–6 wet diapers, and stools daily. The patient was documented as up-to-date with immunizations, including seasonal influenza vaccine, and did not attend daycare. History did, however, include recent sick contact three days prior to initial symptom development and 10 days prior to presentation to the ED with her 5-month-old sister who had self-limited upper respiratory symptoms, which resolved as our patient showed early upper respiratory symptoms.

**Figure 1:**
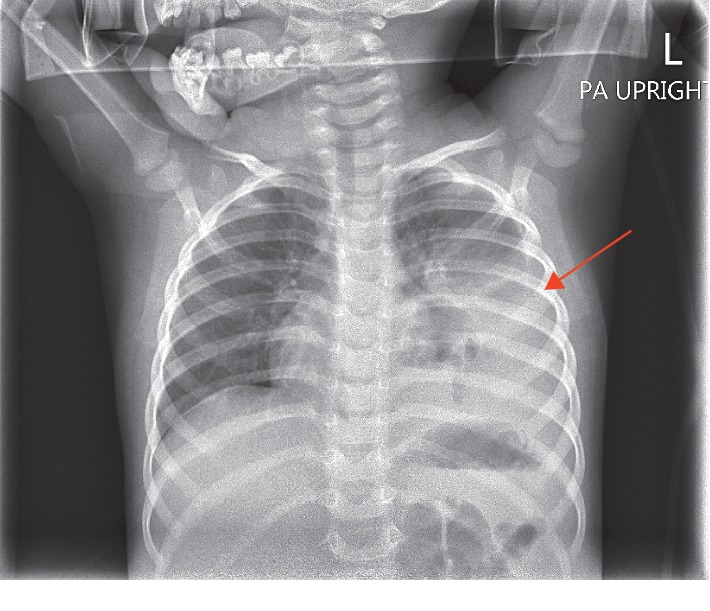
Posterioranterior (PA) chest X-ray demonstrating large consolidation in left lower lobe secondary to pneumonia vs. atelectasis with moderate-sized left pleural effusion noted.

**Figure 2:**
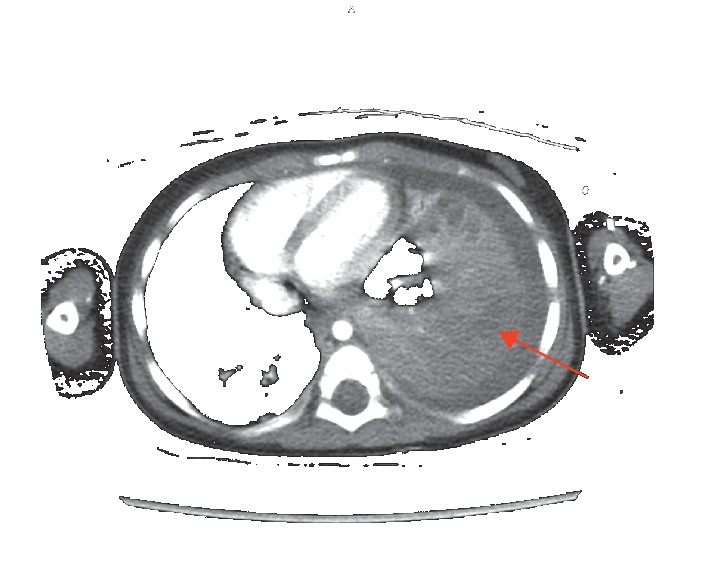
Contrast-enhanced CT of the chest (axial view) demonstrating moderate-to-large left-sided pleural effusion with collapse of left lung and diffuse consolidation involving the entire left lung.

**Figure 3:**
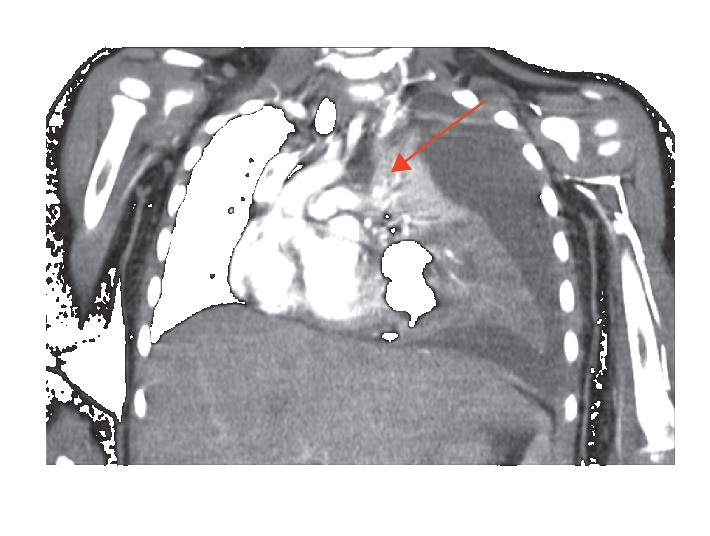
Contrast-enhanced CT of the chest (coronal view) demonstrating mediastinal shift to the right.

On arrival to the ED, the patient's labs were negative by polymerase chain reaction (PCR) for influenza A and B, as well as Respiratory Syncytial Virus (RSV) which were all obtained via nasopharyngeal swab 34 minutes after presentation to the ED. Initial complete blood count (CBC) showed a leukocytosis of 21,800/mm^3^ with 67% neutrophils and 31% lymphocytes. Following labs and physical exam findings, the patient met criteria for admission [2] exhibiting tachycardia (HR = 174, nl 80–120), tachypnea (RR = 44, nl 20–28), and grunting on exam. Blood cultures were drawn before the start of empiric antibiotics and showed no growth after 72 hours. Nasopharyngeal swab to screen for MRSA was negative. The patient was given an intravenous fluid bolus, albuterol/ipratropium breathing treatment, a dose of dexamethasone, and ceftriaxone. Her initial CXR showed left lower lobe pneumonia and parapneumonic effusion with a complex left pleural effusion (Figure 1). An ultrasound was ordered due to evidence of pleural effusion on chest x-ray. It showed a small-to-moderate somewhat complex left pleural effusion demonstrating several irregular septations. On hospital day three, subsequent contrast-enhanced CT of the chest confirmed a large left pleural effusion and lobar consolidation with major atelectasis and mediastinal shift (Figures 2 and 3). Given the severity of the midline shift and her decreasing clinical presentation and respiratory status, vancomycin was added to the antibiotic regimen and pediatric surgery was consulted for left video-assisted thoracoscopic surgical (VATS) decortication and chest tube placement.

VATS was performed on hospital day 4. Prior to the surgery, the patient had received 3 days of ceftriaxone 15 mg/kg initial dose in the ED followed by 50 mg/kg/day and 2 days of vancomycin 10 mg/kg Q6H. VATS confirmed empyema with the presence of grossly purulent pleural fluid. In an unforeseen turn of events, VATS decortication was stopped prematurely due to an end-tidal CO_2_ (EtCO2) of 135 mmHg and airway compromise. The endotracheal tube was removed, which revealed a blood clot obstructing the distal end. The patient was reintubated with improvement in air entry and a decrease in EtCO_2_. The procedure was aborted, and she was taken to the PICU for airway management.

An analysis of the pleural fluid revealed an LDH of 989 U/L, protein 3.5 g/dL, pH 7, and fluid RBC 4,000/mm^3^ along with WBC 732/mm^3^ with 83% neutrophils. Cultures from pleural fluid revealed no growth. Repeat CXRs showed persistent infiltrate in the left lower lobe. At this time, dexamethasone was started. The patient was extubated without complication on hospital day 6 and was weaned to room air on hospital day 8. Vancomycin and ceftriaxone were continued. On hospital day 9, the chest tube was removed, but repeat CXR showed persistent pleural fluid with airspace disease. Her clinical appearance appeared to worsen with increased lethargy and repeat tachycardia and tachypnea.

Pleural ultrasound was performed which demonstrated small-to-moderate, somewhat complex left pleural effusion with several irregular septations. On hospital day 10, repeat contrast-enhanced CT of the chest showed a large loculated collection of air and persistent consolidation of the left lower lobe along with persistent rightward shift of the mediastinum. She was taken back to the operating room (OR) for repeat left VATS decortication and reinsertion of the chest tube at which point she was still receiving ceftriaxone and vancomycin. Upon surgical exploration, she was found to have a large left lung abscess, left-sided pneumothorax, and complex pleural effusion. Pleural fluid analysis revealed uptrending LDH 3,761 U/L with RBC 25,000/mm^3^ and WBC 1,587/mm^3^ with 91% neutrophils and a pH of 6.92. Repeat pleural fluid cultures continued to show no growth. Antibiotics were broadened to vancomycin and meropenem. ID was consulted and recommended stopping vancomycin and meropenem along with starting linezolid 140 mg Q8H for 4 weeks on hospital day 13. The patient began to show clinical improvement.

Seven days after the second VATS, a Heimlich valve was placed onto her chest tube which demonstrated persistent air leak. CXR showed persistent left airspace disease and consolidation. Respiratory pathogen panel nasopharyngeal swab (ordered on hospital day 10) detected positive for only rhinovirus/enterovirus. Leukocytosis resolved to 12,560/mm^3^. The patient was sent home on hospital day 18 with the chest tube and Heimlich valve in place and plan to follow-up with outpatient pediatric surgery in one week. A referral to outpatient pediatric pulmonology was also scheduled.

The patient received Home Health Care (HHC) for dressing change and monitoring. She was seen by pediatric surgery one week after discharge where she obtained follow-up CXR. The chest tube was reinforced after lower stitch detached. She continued with weekly CBC orders and linezolid therapy. Labs continued to normalize.

The young, malleable patient was followed up with pediatric pulmonology at a tertiary academic institution 14 days after discharge to evaluate pulmonary function following chest tube placement. On this first visit, the chest tube was removed. The same day, ultrasound indicated findings suspicious for left diaphragmatic paralysis. Chest fluoroscopy showed findings consistent with left diaphragmatic paralysis from possible phrenic nerve damage with paradoxical motion, and CXR showed consistent elevation of the left hemidiaphragm and trace left-sided pleural effusion. There was no evidence of pneumothorax or focal air space consolidations. The patient was scheduled for a 3-month follow-up and CXR.

The patient's family established care with a pediatric provider for routine health maintenance and management of iron deficiency (anemia) diagnosed during hospitalization. Labs continued to normalize with WBC <8.0 and Hb of 9.4.

On the 3-month follow-up with pediatric pulmonology, it was noted that the patient had possible ptosis of the left eye with pupillary miosis. Pulmonology noted that, it was possible that this can occur due to contact of the chest tube with the stellate ganglion near the apex of the left lung.

Respiratory function continued to improve, and the patient was recommended for immune deficiency evaluation.

## 3. Discussion

This case overviewed an otherwise healthy pediatric patient with complicated CAP, subsequent empyema, and inability to isolate a causative organism. The patient presented with single occurrence of seizure-like activity and prolonged cough, fever, and dyspnea with the only organism identified as rhinovirus/enterovirus. *S. pneumoniae*, followed by *S. pyogenes* and *S. aureus*, remain the most commonly identified species as the cause of common complications of pneumonia in children [3]. This patient, however, repeatedly tested negative for bacterial etiologies of parapneumonic effusion, heightening our suspicion for viral cause of pneumonia and possible primary immune-deficiency.

### 3.1. Rhinovirus Pneumonia in the Pediatric Patient

With pneumonia being a leading cause of pediatric hospitalization in the United States [4], multiple studies have investigated the etiology of pediatric pneumonia in varying ages and settings, with various microbiologic techniques. Studies continue to demonstrate that rhinovirus should not be ignored as a common microorganism associated with CAP.

A 2015 prospective, multicenter, population-based, active surveillance study by Jain et al. [5] investigated the incidence and causes of CAP requiring hospitalization amongst children in the United States. Bacterial pathogens were considered to be present if detected in blood, endotracheal aspirate, nasopharyngeal or oropharyngeal swab, bronchoalveolar lavage, or pleural fluid via culture or PCR. Similarly, viral pathogens were present if detected in a nasopharyngeal or oropharyngeal swab via PCR. Of the 2,358 children who had evidence of pneumonia, the most commonly detected pathogens were viruses. Specifically, rhinovirus was the second most common detected pathogen (in 27% of children), second only to respiratory syncytial virus (RSV). Furthermore, the incidence of RSV, rhinovirus, adenovirus, influenza, coronavirus, human metapneumovirus, and *S. pneumoniae* were higher amongst children younger than 5 years of age and highest amongst children younger than the age of 2, like our patient. However, for due diligence, we recognize that rhinovirus was also detected in 17% of controls in comparison to 22% of children with pneumonia. Due to the ability of rhinovirus to shed up to more than 2 weeks after infection, it can be difficult to interpret its detection in pediatric pneumonia, specifically in asymptomatic children [5]. Furthermore, Esposito et al. collected data from children with radiographically confirmed CAP in whom 17 respiratory viruses were detected in respiratory secretion samples using the Luminex xTAG Respiratory Virus Panel (RVP) Fast assay [6]. The results of this large study found RSV and rhinovirus to be the most common pathogens associated with CAP in single viral infections and coinfections. They were isolated in more than 50% of cases, emphasizing a possible role in pediatric CAP.

Clinical characteristics of rhinovirus-associated pneumonia were also observed in a retrospective study by Hartiala et al. [7]. Of the 313 children with pneumonia, rhinovirus was detected in 82. The characteristics of children with rhinovirus-associated pneumonia were younger age (less than two years old), history of preterm birth, and higher median white blood cell count at presentation. Rhinovirus-associated pneumonia presented as a more severe disease with higher inflammatory markers and clinical presentation that required intensive care. Increased levels of white cell counts have been earlier reported in rhinovirus-related lower respiratory tract infections [8]. However, inflammatory markers are not highly specific in differentiation between viral versus bacterial cause of infection, and studies may benefit from the use of better biomarkers or microbiologic methods to confirm or exclude bacterial coinfection in rhinovirus-associated CAP.

### 3.2. Our Suspicion for Rhinovirus

Due to the clinical ambiguity surrounding the diagnosis of bacterial versus viral pneumonia, we wish to address the rationale for considering rhinovirus as the causative microorganism in this case. From the beginning, our approach followed the national guidelines for the management of CAP by obtaining blood cultures after our patient met criteria for hospitalization [2]. The first set of cultures was drawn 34 minutes after arrival to the ED and 90 minutes prior to the start of ceftriaxone. While it is true that an initial negative blood culture does not rule out a bacterial cause [9], traditionally, negative blood cultures assist in narrowing down the differential to a possible viral cause of pneumonia. Though this method is not without error, it is acceptable because strict diagnosis for viral pneumonia continue to be vague [10]. Two negative blood cultures in addition to a negative wound culture, two negative cultures of pleural fluid, and two negative nasopharyngeal swab cultures expanded the realm of viral causes for our patient. In addition, as per the patient's parents, the patient did not receive antibiotics at any time prior to ED admission, so we do not have concerns that her cultures would be altered for that reason. Additionally, we understood that residual positivity rate after starting empiric antibiotics in septic patients remained high enough that cultures are worthwhile [11], so we continued to order them. Fritz et al. commented on factors that contribute to a low prevalence of bacteremia in pneumonia among pediatric patients. The factors include an “increased burden of viral etiologies of pneumonia” with reference to Jain et al. [12] as previously mentioned. In their 2015 study, 44% of detectable pathogens were viral and only 8% were bacterial.

Finally, the patient's seizure-like activity increased our clinical suspicion for a possible underlying viral etiology. Febrile seizures are a clinical diagnosis with still unknown exact etiology; however, even so, Choi et al. [13] noted that high levels of proinflammatory cytokines released during viral infection may be linked to the onset of febrile seizures in pediatric patients, likely because of the still developing brain. Additionally, it is worth noting that oseltamivir has been shown to be weakly cytotoxic [14]; regardless of when our patient took oseltamivir, it was unlikely to have had any contribution to the onset of seizure-like activity.

Given our patient's age, recent sick contact, a positive respiratory panel for only rhinovirus/enterovirus, and febrile seizure-like activity prior to admission, it is possible that the presentation and preliminary labs are due to an underlying viral infection with rhinovirus/enterovirus alone.

### 3.3. Management of Empyema

The management of complicated pneumonia in pediatric patients is an area of controversy. Different approaches are generally categorized as conservative, involving the use of antibiotics alone versus procedural interventions to drain the pleural space, as performed in our case. Antibiotics are a critical component in medical management for parapneumonic effusion and empyema [4]. Regimens are guided by local policies surrounding susceptibility patterns focusing on the current, increased resistance of *S. pneumoniae* and MRSA to penicillin. As seen in our case, it is crucial to always attempt to obtain blood cultures prior to initiation of antibiotics. In absence of confirmed organism by culture, studies suggest empiric coverage with ceftriaxone or cefotaxime [1]. Some experts suggest the addition of clindamycin for broader coverage of anaerobic or MRSA coverage, with the substitution of vancomycin for clindamycin for suspected MRSA pneumonia. Antibiotics would be switched to oral once drainage is complete and patient improves without oxygen, generally three to four weeks in duration given the absence of additional complications.

### 3.4. Procedural Intervention in Management of Empyema

Various procedural interventions are available for pediatric patients with empyema such as small bore percutaneous chest tube placement (with or without fibrinolytics), thoracentesis, video-assisted thoracoscopic surgery (VATS), and open thoracotomy with decortication. Initially, diagnosis and the decision to drain a pediatric patient with pleural effusion were based on the staging of pleural infection via thoracentesis [15]. However, now thoracentesis is not routinely used in all patients. VATS is performed under general anesthesia with the patient in a supine position and affected side of the chest elevated at 45 degrees to provide adequate exposure and prevent secretion overflow. A single endotracheal tube is used. The first trocar is placed using an open technique in the 5^th^ or 6^th^ intercostal space, midaxillary line. Continuous flow of carbon dioxide is used for lung deflation. There is a risk of displacement of the mediastinum, which can lead to impaired oxygenation, decreased venous return to the heart and carbon dioxide retention. This is especially concerning in small children, such as the patient in this case, due to the mediastinum being displaced more easily [16]. The benefits of VATS, however, outweighed the risk in this case since the patient already experienced mediastinal shift from the progressing infection. VATS, therefore, became critical for resolution of the ravaging infection. Several recent studies suggest that VATS and fibrinolytics decrease the need for reintervention and length of hospitalization, as well as improve health outcomes in comparison with tube thoracostomy alone [17, 18].

Several pediatric studies of empyema management demonstrate that the use of VATS results in earlier, more complete resolution of the infection and decreased hospitalization in comparison with chest tube drainage and medical management [17]. When VATS was compared with fibrinolytic agents as initial therapy for empyema in pediatric studies, there was no significant difference in length of hospital stay, failure rate, days of oxygen requirements, analgesic requirement, and radiologic outcomes [18, 19]. Our patient's presentation provided a clear need for procedural intervention that ultimately cleared enough of her rampant infection, whether bacterial or viral, to allow for complete, eventual resolution of the infection. While she has experienced sequalae that will continue to be monitored, the benefits of these procedures certainly outweighed the cost in her case.
